# Comparative effectiveness and economic evaluation of Chuna manual therapy for chronic neck pain: protocol for a multicenter randomized controlled trial

**DOI:** 10.1186/s13063-018-3016-6

**Published:** 2018-11-29

**Authors:** Ho Jeong Do, Joon-Shik Shin, Jinho Lee, Yoon Jae Lee, Me-riong Kim, Jae-Heung Cho, Koh-Woon Kim, In-Hyuk Ha

**Affiliations:** 1 Jaseng Hospital of Korean medicine, 536 Gangnam-daero, Gangnam-gu, Seoul, 06110 Republic of Korea; 2 Department of Korean Rehabilitation Medicine, College of Korean Medicine, Kyung Hee University, Seoul, 02447 Republic of Korea; 3 Jaseng Spine and Joint Research Institute, Jaseng Medical Foundation, 3F JS Tower, 538 Gangnam-daero, Gangnam-gu, Seoul, 06110 Republic of Korea

**Keywords:** Neck pain, Spinal manipulation, Cost-benefit analysis, Complementary therapies, Protocol

## Abstract

**Background:**

Neck pain is a highly prevalent medical condition that incurs substantial social burden. Although manual therapy is widely used for treatment of neck pain, the body of evidence supporting its effectiveness and safety is not conclusive. The aim of this study is to examine the effect, safety, and cost-effectiveness of Chuna manual therapy, a traditional Korean manual therapy for treatment of various musculoskeletal complaints.

**Methods/Design:**

This study is the protocol for a two-armed parallel, assessor-blinded, multicenter, randomized controlled trial. A total 108 patients with chronic neck pain (time to onset ≥ 3 months, numeric rating scale [NRS] of neck pain ≥ 5) will be recruited at five Korean medicine hospital sites. Participants will be allotted to one of two groups (*n* = 54, respectively): the Chuna manual therapy group, and the usual care (conventional physical therapy and medication treatment) group. Ten sessions of Chuna manual therapy or usual care will be administered twice a week for five weeks. Since the study design does not permit patient or physician blinding, the outcome assessor and statistician will be blinded. The primary outcome will be the visual analogue scale (VAS) of neck pain at 5 weeks after randomization. Secondary outcomes include the VAS of radiating arm pain, NRS of neck pain and radiating arm pain, Vernon-Mior neck disability index (NDI), Northwick Park neck pain questionnaire (NPQ), EuroQol-5 Dimension (EQ-5D), EQ-VAS, patient global impression of change (PGIC), economic evaluation, adverse effects, and drug consumption. Follow-up outcome assessments will be conducted at 3, 6, 9, and 12 months after randomization.

**Discussion:**

This study will evaluate the comparative effectiveness of Chuna manual therapy and usual care on chronic neck pain. Adverse events, and costs and effectiveness (utility) data will be evaluated to assess safety and exploratory cost-effectiveness (economic evaluation). This study aims to provide evidence on the effectiveness, safety, and cost-effectiveness of Chuna manual therapy.

**Trial registration:**

Clinical Research Information Service (CRIS), KCT0002732. Registered on 13 March 2018. ClinicalTrials.gov, NCT03294785. Registered on 27 September 2017.

**Electronic supplementary material:**

The online version of this article (10.1186/s13063-018-3016-6) contains supplementary material, which is available to authorized users.

## Background

### Background and rationale

Neck pain is generally defined as pain in the posterior neck region from the superior nuchal line to the spinous process of the fifth thoracic vertebra [1]. Neck pain is the fourth major cause of years lived with disability and average prevalence is estimated at > 30% [2, 3], while lifetime prevalence estimates are put as high as 66% [4]. Neck pain is the cause of varying degrees of disability and incurs high medical expenses through increased use of medical services, sick leave, and lost productivity [5–8]; the heavy social burden it imposes points to the pressing need for timely and effective management of neck pain to cut related personal and social costs [9].

Spinal manual therapies are among the most frequently used treatments for neck pain along with medication and kinesiotherapy [1]. Chuna manual therapy is a specialized type of manual therapy where the practitioner uses manual and/or physical force with optional devices to apply appropriate correcting force to specific body areas to treat various dysfunctions and pathophysiologic conditions [10]. Current Chuna manual therapy effectively combines traditional Chuna, mainly derived from traditional Korean medicine, with the strong points of Chinese, Japanese, and American manual therapies, to treat musculoskeletal and neuromuscular diseases including various cervical disorders [11].

While a multitude of studies examining the effect of various manual therapies including Chuna manual therapy on neck pain have been conducted, the quantity and quality of previous trials have been found lacking and have thus failed to reach a solid conclusion. A 2015 Cochrane review [12] on manipulation for neck pain analyzed a total of 51 randomized controlled trials (RCTs) (2920 study participants total) as of November 2014 and reported that multiple sessions of cervical manipulation were more effective than medication for pain management and functional recovery in acute and subacute neck pain in the intermediate or long term. However, there was a distinct paucity of studies comparing the effect of manual therapy with active controls for chronic neck pain, only yielding RCTs comparing high-dose versus low-dose manipulation and heterogeneous studies in acute, sub-acute, and chronic pain patient populations. Another recent systematic review of RCTs on manual therapy for neck pain called into question the specificity of the diagnostic criteria and treatment interventions employed [13]. Moreover, cost-effectiveness, in addition to effectiveness, should be given due consideration as it is of heightened importance to patients, practitioners, and health policy makers when determining optimal treatments and usual care for chronic neck pain.

Furthermore, few studies have examined the adverse effects of Chuna manual therapy for neck pain. A comprehensive 2010 Cochrane systematic review on manipulation and mobilization for neck pain concluded that existing manipulation and mobilization studies fail to provide a systematic reporting system for adverse events (AEs) [14]. Considering that a previous study on the frequency and characteristics of side effects of spinal manipulation reported that up to half of patients receiving manipulation presented with adverse effects lasting ≥ 24 h [15], inclusive studies on the adverse effects of Chuna manual therapy are warranted.

### Objectives

This rigorous clinical trial was designed to the aim of determining the effectiveness, safety, and cost-effectiveness of Chuna manual therapy for chronic neck pain. First, the comparative effectiveness and safety of Chuna manual therapy will be evaluated through comparison of Chuna manual therapy and usual care as assessed using pain and functional indices as well as quality of life and adverse effect measures. In addition, analysis of long-term cost-effectiveness will examine the cost-effectiveness of Chuna manual therapy compared to usual care.

## Methods/design

This multicenter RCT protocol is reported in concordance with the Standard Protocol Items: Recommendations for Interventional Trials (SPIRIT) 2013 Statement (Additional file 1).

### Trial design and study setting

**Fig. 1:**
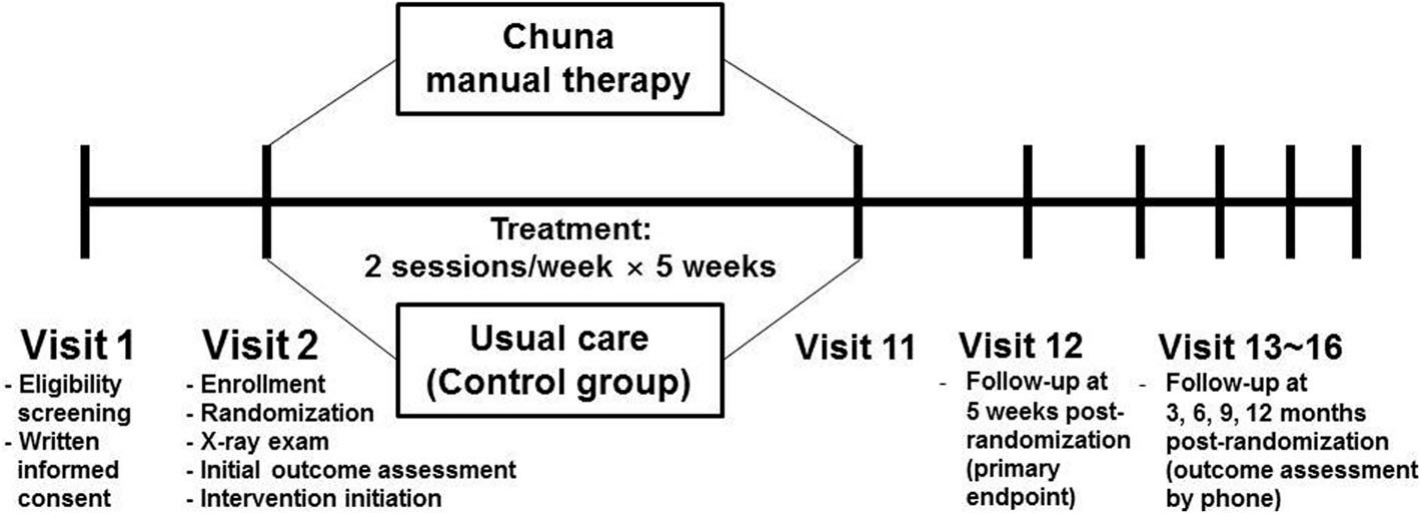
Study flow

**Fig. 2:**
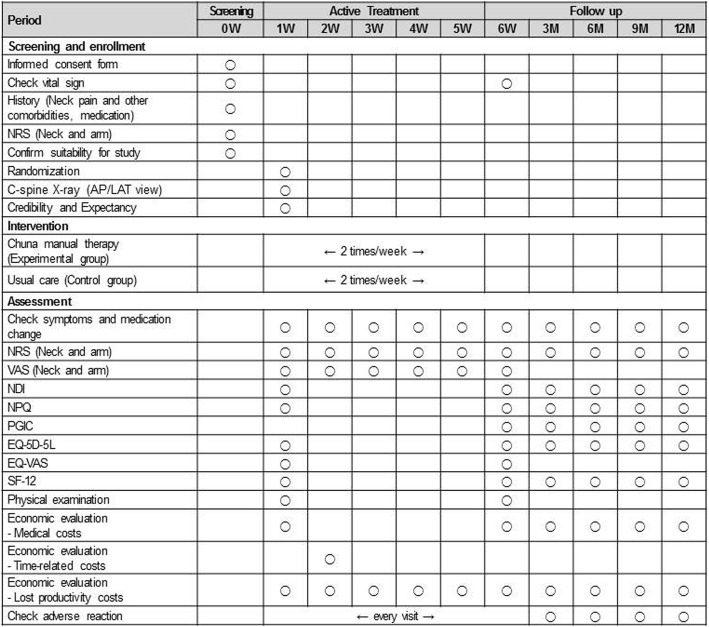
Timepoints of each assessment index

This study is a two-armed parallel multicenter RCT with assessor-blinding. The study participants will be randomly allocated to one of two groups—the Chuna manual therapy group or the usual care group—at a ratio of 1:1. Participants will be recruited at five Korean medicine hospitals in Korea (Jaseng Hospital of Korean medicine sites located in Seoul, Bucheon, Daejeon, and Busan, and Kyunghee University Korean medicine Hospital at Gangdong located in Seoul) from October 2017 to June 2018 (anticipated). Flyers advertising study participant recruitment will be posted within the hospital grounds and on the hospital and external websites. Further information on the medical institutions acting as the study settings recruiting study participants can be found at the following trial registration sites (Clinical Research Information Service [CRIS] KCT0002732 [https://cris.nih.go.kr/cris/search/search_result_st01.jsp?seq=9719] and ClinicalTrials.gov NCT03294785 [https://clinicaltrials.gov/ct2/show/NCT03294785]). A total of 108 participants with pain and/or discomfort of the neck and surrounding area (trapezius muscle area) will be recruited regardless of accompanying radiating arm pain. Participants will receive treatment twice a week over a period of five weeks in the outpatient department. On their first visit, participants will be screened for study eligibility according to study inclusion and exclusion criteria; from the second visit onward, participants will receive the neck pain treatment relevant to their random allocation group. All study interventions, participants, and execution will be managed in accordance with the study protocol (Figs. 1 and 2).

### Participants

#### Inclusion criteria

1. Patients with chronic neck pain (of pain duration of ≥ 3 months);
2. Neck pain patients with or without radiculopathy, with an NRS of neck pain greater than or equal to that of radiating aim pain;
3. Patients with an NRS of ≥ 5 for the past three days;
4. Patients aged 19~60 years;
5. Patients who have agreed to and provided written informed consent for trial participation.

#### Exclusion criteria

1. Patients diagnosed with severe pathologies which may be the main cause of neck pain (e.g. spinal metastasis of tumor, acute fracture, spinal subluxation);
2. Patients with a medical history of cervical spine surgery;
3. Patients with concurrent chronic conditions that may interfere with interpretation of treatment effects, safety, or other outcomes (e.g. chronic renal failure, vertebral artery complications, rheumatoid arthritis, Down syndrome);
4. Patients with severe concurrent neurologic symptoms such as progressive neurologic deficit or spinal cord injury;
5. Patients suffering severe neuropsychological disease;
6. Patients with concurrent medication intake of steroids, immune-suppressants, neuropsychological medicine, or other medication that may affect study results;
7. Patients who have received Chuna manual therapy or physical therapy or taken medication such as non-steroidal anti-inflammatory drugs (NSAIDs) that may potentially affect pain levels within the past week;
8. Current pregnancy or planning for pregnancy;
9. Patients who are participating in other studies or who are otherwise unsuitable for participation in the clinical trial as determined by the researchers.

### Randomization and allocation concealment

Stratified block randomization will be conducted with stratification by each study site to help control for potential bias in the control and experimental groups. Upon randomization within each study site, participants will be allocated to either the control or experimental group according to the allocation code, and the number of participants in each group will be identical between groups for all study sites.

Random allocation will be performed by a statistician by randomly allocating 54 participants to each group under the condition where the possibility of being chosen is identical for all individuals using SAS version 9.4 statistical package (SAS institute, Cary, NC, USA). The number of participants for each study site will be 24 for Jaseng Hospital of Korean medicine Gangnam branch, 22, respectively for Jaseng Hospital of Korean medicine Bucheon, Daejeon, and Haeundae branches, and 18 for Kyunghee University Korean medicine Hospital at Gangdong. The results of random allocation will each be sealed in opaque envelopes before being sent to each study site and will be kept stored in double lock cabinets.

The researchers responsible for registration of eligible participants at each study site will fully explain to the participants about the clinical trial before receiving written informed consent from the participants. The coordinators will assign randomized numbers by the sequence of registration to participants to be included in the clinical trial following the inclusion/exclusion criteria who will thus be allocated to either one of the two groups at a 1:1 ratio by opening the sealed envelope in front of the participant. The opened envelopes will be kept separately in a double-lock cabinet. The randomized numbers allocated to each participant will be recorded in the electronic medical chart records.

### Blinding

Since it is not feasible to double-blind the practitioner and participant regarding allocation to the two types of treatment due to the study design, blinding of the outcome assessors and statistician will be performed. The outcome assessor will not partake in treatment and a clinical research nurse or a Korean medicine doctor blinded to group allocation will evaluate the participant in a separate area before treatment, and will not try to guess the treatment allocation group to which the participant may be allocated. Computerized data passed on to the statistician will not contain any information identifying patient allocation to either of the two groups.

### Interventions

#### Chuna manual therapy

Chuna specialists convened to discuss Chuna techniques not only directed at the cervical region for treatment of neck pain but also the thoracic, lumbar, and sacroiliac regions (i.e. Chuna techniques for the cervical, thoracic, and lumbar spine, pelvis, sacrum, and pubic and hip joints). A list of Chuna manual therapy techniques was compiled; after specialist review, a final list was decided on and confirmed. This list for Chuna manual therapy is based on “*Chuna manual medicine* (2.5nd edition)” that was published by the Korean Society of Chuna Manual Medicine for Spine and Nerves and is used as a textbook at the Schools and Colleges of Korean medicine in Korea [16] (Table 1). Chuna manual therapy was administered by Korean medicine doctors with ≥ 3 years of clinical experience of Chuna; to minimize the difference in styles of Chuna techniques, educational training sessions were conducted to standardize the administration methods of different Chuna techniques. The Korean medicine doctor administering Chuna manual therapy first assesses patient symptoms and conducts physical examination and radiographic examination prior to treatment. The total number of Chuna techniques used within the list was not limited; the types of Chuna techniques used for each session of treatment will be recorded in the electronic medical chart records.

**Table 1:** Semi-standardized treatment plan of Chuna manual therapy techniques to be used in the study

Region	Chuna manual therapy	
Cervical spine	Distraction	Supine position cervical distraction method using towel Supine position cervical distraction method Prone position cervical distraction method
	Correction	Supine position cervical correction method Supine position cervical JS distraction and correction method Supine atlas correction method Supine occipital correction method
Thoracic spine and thoracic cage	Upper thoracic vertebral fascial Chuna	Seated position upper thoracic extension dysfunctionmuscle relaxation/strengthening method Seated position upper thoracic flexion dysfunctionmuscle relaxation/strengthening method Seated position upper thoracic neutral dysfunction muscle relaxation/strengthening method
	Middle and lower thoracic vertebral correction	Supine thoracic extension dysfunction correctionmethod Prone both hands pisiform lower thoracic flexion dysfunction correctionmethod
	Lower thoracic vertebral fascial Chuna	Seated position lower thoracic extension dysfunctionmuscle relaxation/strengthening method Seated position lower thoracic flexion dysfunctionmuscle relaxation/strengthening method Seated position lower thoracic neutral dysfunction muscle relaxation/strengthening method
	Thoracic cage fascial Chuna	Seated position first rib elevation dysfunction muscle relaxation/strengthening method
	Thoracic cage joint mobilization	Supine position second rib lateral flexion dysfunction joint mobilization method
Lumbar spine	Lumbar distraction	Prone position lumbar-sacral junction distraction method Side-lying position lumbar distraction method
	Lumbar correction	Side-lying position lumbar extension dysfunction correction method Side-lying position lumbar flexion dysfunction correction method Side-lying position lumbar neutral dysfunction correction method
	Lumbar fascial Chuna	Seated position lumbar bilateral flexion dysfunction muscle relaxation/strengthening method
	Motion Style Acupuncture Treatment (MSAT)	Quadratus lumborum muscle MSAT Iliopsoas muscle MSAT
	Vertebral flexion/distraction	Flexion, distraction Lateral flexion, side bending Circumduction Circulation of the foramen magnum Extension
Pelvis	Iliac correction	Prone position leg raising iliac correction method Prone position iliac anterior rotation correction method Prone position inflare/outflare adjustment method using the pisiform and metacarpophalangeal joint of the index finger Prone position iliac posterior rotation/sacral lateral bending correction method Side-lying position iliac correction method
Sacrum	Sacral correction	Prone position sacral flexion dysfunction correction method Prone position sacral extension dysfunction correction method Prone position sacral lateral rotation dysfunction correction method Side-lying position sacral dysfunction correction method
Pubis	Pubic distraction	Supine position pubic distraction method
	Pubic correction	Supine position superior pubic shear correction method Supine postion inferior pubic shear correction method
Hip joint	Hip joint mobilization	Supine position hip joint mobilization method – joint play method, Dong-Qi therapy

Chuna manual therapy is a therapy that can be largely characterized by high velocity low amplitude thrusts and spinal mobilization. Thrusts are of high velocity and low amplitude directed at the spinal joints, slightly over the passive range of motion. Spinal mobilization does not involve thrusts and only applies repetitive manual force to the spinal joints within the passive range of motion [17].

A total of 10 sessions of Chuna manual therapy will be conducted twice a week for five weeks. On the first visit, about 20 minutes will be allotted for diagnosis with about 10 minutes set for treatment. For return visits, about 10 minutes will be allotted for diagnosis and treatment, respectively.

#### Usual care

Usual care consists of oral medication and physical therapy where a total of 10 sessions of physical therapy will be administered twice a week for five weeks. Physicians will be provided with lists of high frequency medication and physical therapy prescriptions for non-specific neck pain, cervical disc herniation, and sprain and strain of the neck extracted from the 2014 National Patient Sample of the Korean Health Insurance Review & Assessment Service (HIRA-NPS) to take into consideration. All types of medication and physical therapy used will be recorded in a separate electronic case report form (CRF) to maintain assessor blinding (Additional file 2).

#### Co-interventions

No additional treatment (Korean medicine treatment, conventional medicine medication or procedures, physical or exercise therapy) to the purpose of relieving pain other than the treatment provided in the study will be allowed over the course of treatment until the sixth week which is the primary endpoint. However, the research will set no limits after the sixth week. In case the participants in the experimental or control groups experience severe pain during the study period, administration of acetaminophen (maximum dosage of 4 g per day) will be allowed as a rescue medication due to ethical issues. Its use will be specifically reflected and assessed in describing outcomes in the results paper.

### Outcomes

#### Primary outcome measurement

The primary outcome will be the visual analogue scale (VAS) of the past three days’ neck pain. VAS refers to an evaluation tool that records the degree of pain the patient feels with one end indicating no pain and the other end indicating the most severe pain imaginable on a 100 mm line [18, 19]. Participants will be asked to mark the one point that best describes his or her neck pain during the previous week.

The difference in VAS at baseline (visit 2) and the primary endpoint (follow-up 1 [visit 11]: five weeks after visit 2) will be used to compare the two groups to identify the difference in effect size.

#### Secondary outcome measures

The degree of radicular pain during the past three days will be evaluated with VAS. The degree of neck pain and radiculopathy during the past three days will also be evaluated with the Numeric Rating Scale (NRS). The NRS is a numeric pain index that objectifies subjective pain. Subjects are asked to select from 0, indicating no pain, to 10, indicating the most severe pain one can imagine [20, 21]. The functional condition of the neck will be evaluated using the Vernon–Mior Neck Disability Index (NDI) questionnaire. The NDI, which was designed to investigate the degree of neck disability calculates the average value by dividing the total score by the number of answered items. It is composed of ten items in questionnaire format with each item scored with 0~5 points, adding up to 50 points in total. Higher scores indicate higher levels of disability. The Northwick Park Neck Pain Questionnaire (NPQ) will also be used to evaluate the degree of functional disability due to neck pain. The NPQ is a self-report evaluation tool for assessment of subjective neck pain and its impact on everyday life. It is composed of nine items, each consisting of five questions, evaluating symptoms in terms of functional disability over the past three days. Each item is scored with 0–4 points, where 4 points indicates the severest functional disability; the total score is calculated as the sum of scores from the nine questions.

For comprehensive evaluation of improvement in neck pain and related functional disability, the patient global impression of change (PGIC) will also be investigated [20, 22]. The PGIC evaluates the degree of patient improvement in seven levels: 1, very much improved; 2, much improved; 3, minimally improved; 4, no change; 5, minimally worse; 6, much worse; and 7, very much worse. The evaluation index was originally developed for psychological purposes but is now also variously used in other medical fields for evaluation of improvement in pain.

Physical examinations will be performed to evaluate for pain upon movement of the neck. Upon registration in the study (visit 2), existence of pain on flexion, extension, lateral bending, and rotation will be identified, and it will also be investigated identically at the primary endpoint. Muscular weakness and sensory loss will also be assessed upon registration and at the primary endpoint.

Evaluation tools for the quality of life of participants will be the five-level version of EuroQol-5 Dimension (EQ-5D-5 L), EuroQol Visual Analogue Scale (EQ-VAS), and the 12-Item Short-Form Health Survey (SF-12).

The EQ-5D-5 L is a tool developed to measure health-related quality of life, and is widely used in the healthcare field. EQ-5D-5 L is composed of five items inquiring into current health status (i.e. mobility, self-care, usual activities, pain/discomfort, and anxiety/depression) and each item evaluates functions in five levels (level 1, no problems; level 2, slight problems; level 3, moderate problems; level 4, severe problems; level 5, extreme problems) [23]. In this study, weighted health-related qualify of life scores were calculated by applying a weighted model as estimated in Koreans [24].

The EQ-VAS is an evaluation tool that records the subjective health status of the patient on a 100-mm vertical line with labels for worst health status at one end of the scale and the best imaginable health status at the other end [23]. Subjects are asked to mark a point that they feel best represents their health status on that day.

SF-12 is a summarized version of the Short Form-36 Health Survey (SF-36), which is widely used in the evaluation of health-related quality of life [25]. The SF-12 is composed of four items pertaining to physical health and four items pertaining to mental health, with each item containing one or two questions. For physical health, there are two questions on “physical functioning,” two questions on “limitation to roles from physical problems,” one question on “bodily pain,” and one question on “general health condition.” For mental health, there are the following: one question on “vitality (energy/tired);” one question on “social functioning;” two questions on “role limitation from emotional problems;” and two questions on “mental health (mental stress and mental happiness).”

##### Investigation for cost data

In the healthcare field, cost items can be categorized into medical costs, non-medical costs, and costs from productivity loss [26–28]. Medical cost refers to expenses spent in using services at medical institutions (direct medical costs) and unofficial expenses spent in buying health products or medical devices (indirect medical costs). Non-medical costs include costs accompanied in medical service use such as transportation fees, patient time, and caregiver expenses. Cost of productivity loss is the cost of economic losses from not being able to participate in labor due to the disease itself or premature death from disease. The Work Productivity and Activity Impairment questionnaire (WPAI) will be used to calculate the cost of productivity loss and it will be applied for cost–utility analysis.

##### Credibility and Expectancy Questionnaire

The Credibility and Expectancy Questionnaire [29], which consists of a 9-point Likert scale, will be used for the evaluation of participants’ expectations towards treatment. On the first visit of week 1 (visit 2), participants will choose the score that best answers the following question (1 = not at all, 5 = somewhat, and 9 = very much): “How much do you expect the treatment(s) you will receive during the study period to improve your symptoms?”

##### Drug consumption

All medications taken over the study period as rescue medication or prescribed for treatment of the current complaint and their dosage will be recorded through surveys conducted on participant visits. Other physical therapies or injection therapies will also be recorded as the number of treatment sessions.

### Sample size

1. Calculation of number of participants required for the clinical trial: for calculation of significant number of participants, the following is hypothesized based on the previous literature:
  1. Level of significance, α = 0.05;
  2. Type II error (β) is set at 0.2 with power of the test set at 80%;
  3. According to a previous clinical study using VAS as the main assessment tool for Chuna manual therapy in neck pain patients, the effect size of Chuna manual therapy in comparison to the control can be considered to be 1.03 [30], and 1.49 [31], respectively;
  4. Compliance rate will be set at 85% (the predicted rate of receiving ≥ 6 sessions of treatment during the five-week treatment period);
  5. For calculation of sample size, G*Power 3.1.7 was used. With evidence from item (3), application of mean ± SD resulted in conservatively hypothesizing the effect size of this clinical trial as 0.6 because of the high clinical heterogeneity of manual therapies such as Chuna. Application of items (1) and (2) and the effect size resulted in the calculation of a sample size of 90 participants (45 in each group).
2. The mean comparison of pain requires a total of 90 participants (45 in each group), which is the minimum number of participants needed for testing the abovementioned hypothesis. Considering that 15% of participants received < 6 sessions of treatment within the five-week treatment period, a total of 108 participants, 54 in each group, will be extracted for the study.

### Statistical analysis

The main evaluation method will be by intention-to-treat (ITT) analysis that evaluates participants who received at least one session of treatment. Per-protocol (PP) analysis will also be performed, which analyzes only participants who complete participation in the clinical trial in the initial allocated group, excluding participants who withdrew or crossed over during the study. Missing values will be processed mainly by multiple imputation, and last observation carried forward (LOCF) will be used for sensitivity analysis. Participants who received ≥ 6 sessions of treatment during the five-week treatment period will be analyzed separately.

The sociodemographic characteristics and treatment expectancy of participants will be evaluated by group. Continuous variables will be presented as average (standard deviation) or median values (quartile), and the two groups will be compared using Student’s t-test. Wilcoxon rank-sum test will be conducted if the data do not follow normal distribution. Categorical variables will be presented as frequency values (%), and Chi-square test or Fisher’s exact test will be performed as appropriate.

The efficacy variable for this clinical trial is the difference in continuous outcomes (i.e. NRS, VAS, NDI, NPQ, EQ-5D-5 L, SF-12) between baseline and predetermined timepoints. The primary endpoint is one week after completion of treatment (seven weeks after random allocation). Baseline values of each outcome and covariant factors which show statistical difference between groups at baseline will be set as covariates, and analysis of covariance (ANCOVA) with the treatment groups designated as the fixed factor. In addition, to examine the trend difference by visit, repeated measure (RM) ANCOVA will be performed.

To compare the total difference in outcome between the two groups within the treatment period (six weeks) and total study period (one year), the area under the curve (AUC) by specific timepoint after random allocation will be calculated and compared using the Student’s t-test.

Specific timepoints where the VAS and NRS fall under the half line point of the baseline standard will be performed for a comparative analysis on patient improvement rate. To measure the time between random allocation to the point when neck pain improves to less than half the original pain intensity, Kaplan–Meier survival analysis will be used for analysis and the curve will be compared using the log-rank test. A Cox model will be used to calculate the hazard ratio to compare the speed at which pain improves to less than half the original pain. Whether the recovery speed differs by subgroup will also be examined.

#### Subgroup analysis

- Sociodemographic characteristics: body mass index, age (≥ 40 years; and < 40 years), sex, recommendations for surgery, driving, headache, depression, number of sick leave days from work;
- Physical examination: neurologic examination (sensory loss, muscle weakness), pain upon range of motion (ROM) (i.e. flexion, extension, lateral bending, rotation);
- Pain characteristics: area of neck pain (bilateral/unilateral, central/trapezius/back), existence of radiating pain (yes/no, unilateral/bilateral, pain under the elbow joint, pain in the back), pain characteristics (acute onset versus slow onset, persistent versus aggravating), conditions of pain aggravation (understress, fatigue), nocturnal pain;
- Treatment prior to study: experience of Chuna manual therapy, continuation of physical therapy or conventional medication;
- Expectancy and preference for treatment: credibility and expectancy, preference (Chuna manual therapy; usual care; or no preference);
- X-ray examination: head forward posture, lateral deviation of spine, cervical intervertebral disc space, degenerative change;
- Index severity evaluation: VAS ≥ 7 and < 7, NDI high/low.

All statistical analyses were performed with SAS version 9.4 statistical package (SAS Institute, Cary, NC, USA), with the level of significance set at *p* < 0.05.

### Economic evaluation

Economic evaluation will be performed simultaneously with the clinical study to determine the cost-effectiveness of Chuna manual therapy and usual care. The primary economic outcome will be cost per quality-adjusted life years (QALY) gained. The primary economic endpoint will be  set for the study period and for the follow-up period; when estimation for time after this period is needed, it will be by extrapolation of cost and effect using a regression model or secondary analysis that performs decision modeling analysis. Treatment fees from the clinical trial will be calculated by combining the number of treatment sessions and unit fees, where the unit fee will be based on data from national health insurance and fees as charged at the study sites. Estimation of quality of life for calculation of QALYs will use quality of life estimates as deduced by EQ-5D-5 L as the evaluation variant; the AUC method will be used. When the time horizon exceeds 12 months, the fee unit will be standardized to 2017 Korean currency (Won) applying a 5% discount based on the economic evaluation guideline of HIRA. The analytic viewpoint of this study is from a social standpoint; representative values (mean) of the parameters used in the study will be used in baseline analysis. Sensitivity analysis will be performed through probabilistic sensitivity analysis using all possible estimated parameters and representative values.

### Data collection and management

Paper CRFs and electronic CRFs (e-CRFs) utilizing an Internet-based Clinical Research and Trial management system (iCReaT) provided by the Korea Centers for Disease Control & Prevention will be used. Standard operating procedures (SOPs) will be prepared before initiation of the clinical trial. Investigators at supervising organizations will train the outcome assessors and investigators at each study site on how to fill out CRFs, convert electronic data, and adhere to the SOP. CRF data relating to outcome indexes will be entered by double data entry and data entry will be performed at each clinical trial site and supervising sites. After double confirmation of data transfer, all investigators, with the exception of the statistician, will be blocked from data access.

### Adverse events

Adverse events refer to undesirable and unintentional signs (i.e. abnormal laboratory test results), symptoms, or disease occurring after treatment during the process of the clinical trial, which does not have to have a causal relationship with the treatment intervention. Investigators will analyze the frequency of AEs, abnormal laboratory test results, and severe adverse events (SAEs). All description of SAEs will be recorded. AE information will be collected through symptom reports from patients and observations of investigators; occurrence frequency between groups will be analyzed.

Any phenomenon considered to be due to AEs associated with treatment (e.g. symptoms, onset, duration) will be recorded without exception in the investigation forms; what is not recorded will be considered a subjective symptoms. In addition, since the evaluation of AEs may expose the participant allocation group, the evaluation of AEs will be recorded in a separate CRF by the study coordinator; evaluation of the severity of AEs will be based on the evaluation standard for AE severity. Assessment of the causal relationship with treatment will follow the WHO-UMC causality scale and will be categorized into six steps for evaluation (1, definitely related; 2, probably related; 3, possibly related; 4, probably not related; 5, definitely not related; and 6, unknown). After consultation with the study investigators on abnormal laboratory findings, the degree of subjective/objective symptoms will be divided into three levels as proposed by Spilker et al.: Mild (1), no need for additional procedures and no great interference with the subject’s everyday life (function); Moderate (2), significant interference of the subject’s everyday life (function), probable need for additional procedures but followed by resolution after the procedure; and Severe (3), SAEs calling for advanced procedure, and leaving sequelae) will be applied for evaluation.

The researchers responsible for clinical trial conduction at each study site are obligated to explain the possibility of potential AEs that may occur after treatment intervention to the study collaborators, participants, and/or guardians. Educational training sessions on how to report any symptoms of potential AEs occurring after treatment will be provided. Any local, general, or pathophysiologic symptoms should be recorded and classified by symptom type, onset, severity, additional procedures, progress, and causal relationship with the treatment intervention according to the management standard of the clinical trial and also recorded in the participant CRF. The principal investigators at each study site should describe and evaluate any symptoms that occurred during the study period, and any SAE occurring over the study period should be reported to the Institutional Review Board (IRB) of that study site and the supervising clinical trial site (Jaseng Medical Foundation) for determination of continuation or termination of the study. Additional safety information should be reported periodically until the relevant AE is resolved (i.e. resolution of the relevant AE or unavailability for follow-up). The principal investigators at each study site will adhere to the Declaration of Helsinki when performing the clinical trial.

### Data monitoring and safety monitoring

The safety of participants will be reviewed; the CRFs and evidence documents will be compared for certification of completeness of data.

Monitoring will be scheduled for three timepoints: upon recruitment of study participants; in progress during the study period; and upon completion of the clinical trial. Monitoring sessions will be performed by the personnel responsible for monitoring at the supervising study site.

All AEs reported during the study period will be noted to calculate the incidence of AEs. The proportion of participants with AEs in each group will be calculated and compared using Chi-square test or Fisher’s exact test.

### Stopping rules

It will be noted whether study participants completed the study; when treatment or observation is suspended, the reason will also be recorded. For participants who have been suspended from study, follow-up observations may be continued with the participant’s consent. Circumstances that disable further continuation of study participation include SAEs, detection of disease(s) that may potentially affect the study results, and withdrawal of participant consent for study participation.

## Discussion

In previous studies of the effectiveness, safety, and cost-effectiveness of Chuna manual therapy for chronic neck pain, usual care was selected as the control group. While previous clinical studies only mention the term usual care for the treatment of neck pain [32, 33] or report diverse treatments such as physical therapy, analgesics or anti-inflammatory medication, NSAIDs, consultation with a general practitioner (GP), rest, exercise, education, manipulation, electrotherapy, and acupuncture treatment [34–39]. No specific direction for treatment is suggested and no unified guideline for diagnosis, management, or treatment is set. Therefore, rather than setting the control group at the evidence level of standard care, the researchers selected usual care consisting of treatment methods most frequently used in Korea as the control group. Korea employs a national healthcare social security system that is operated by the government; this is based on the national health insurance services that cover the entire South Korean population and its medical institutions. This system manages the approved medical fees with coverage of most medical conditions including neck pain; the HIRA database provides information on received treatments, medical services, and diagnosis [40]. The physicians administering usual care were provided with the most frequently prescribed types of treatment for neck pain from the 2014 HIRA-NPS data so as to place further external validity in selection of treatment approaches.

This RCT will investigate the comparative clinical effectiveness of Chuna manual therapy, a traditional Korean manual therapy, in comparison with the usual care widely used in Korea. However, by guaranteeing autonomy to the physicians as a pragmatic trial in selection of Chuna manual therapy techniques and usual care treatments, there are potential limitations in evaluating and interpreting the study results. This study will be the first well-designed, rigorous large-scale multicenter RCT, and will monitor safety with cost-effectiveness simultaneously. It is thereby expected to provide high-quality evidence of the safety and cost-effectiveness of Chuna manual therapy.

This study will examine the effectiveness, safety, and cost-effectiveness of Chuna manual therapy which will provide an evidence base for clinical practice guidelines of Korean medicine and data towards strengthening coverage of healthcare services within the national health insurance system. It is also hoped to serve as reference for clinical trials in the study of acute or sub-acute neck diseases indicative of Chuna manual therapy.

### Trial status

The study will be initiated in October 2017 and participant recruitment is expected to be completed by June 2018. The study is expected to be completed by August 2019.

## Additional files

Additional file 1: SPIRIT 2013 Checklist. (DOC 123 kb)

Additional file 2: The high rank lists of medication and physical therapy for non-specific neck pain, cervical disc herniation, and sprain and strain of neck were extracted from the 2014 National Patient Sample of the Korean Health Insurance Review & Assessment Service (HIRA-NPS). **Table S1.** Non-narcotic analgesics use list (Top 10 items). **Table S2.** Narcotic analgesics use list (Top 5 items). **Table S3.** Physical therapy use list (Top 10 items). (DOCX 20 kb)

Additional file 3: Principal investigator (PI) and research physicians at main study site. (DOCX 20 kb)

## Abbreviations

ANCOVA: Analysis of covariance
AUC: Area under the curve
EQ-5D-5 L: 5-level version of EuroQol-5 Dimension
EQ-VAS: EuroQol Visual Analogue Scale
HIRA-NPS: National Patient Sample of the Korean Health Insurance Review & Assessment Service
iCReaT: Internet-based Clinical Research and Trial management system
IRB: Institutional Review Board
ITT: Intention-to-treat
LOCF: Last observation carried forward
NDI: Vernon–Mior Neck Disability Index
NPQ: Northwick Park Neck Pain Questionnaire
NRS: Numeric Rating Scale
NSAID: Non-steroidal anti-inflammatory drug
PGIC: Patient global impression of change
PP: Per-protocol
RCT: Randomized controlled trial
RM ANCOVA: Repeated measure analysis of covariance
ROM: Range of motion
SF-12: 12-Item Short-Form Health Survey
SF-36: Short Form-36 Health Survey
SOP: Standard operating procedure
SPIRIT: Standard Protocol Items: Recommendations for Interventional Trials
VAS: Visual analogue scale
WPAI: Work Productivity and Activity Impairment questionnaire
