# Supplementary material for: Comparative effectiveness and economic evaluation of Chuna manual therapy for chronic neck pain: protocol for a multicenter randomized controlled trial
Source: Trials. 2018 Nov 29;19:663. doi: 10.1186/s13063-018-3016-6 (PMC6267027; doi:10.1186/s13063-018-3016-6)
Supplement: Supplementary file 2 — The high rank lists of medication and physical therapy for non-specific neck pain, cervical disc herniation, and sprain and strain of neck were extracted from the 2014 National Patient Sample of the Korean Health Insurance Review & Assessment Service (HIRA-NPS). Table S1. Non-narcotic analgesics use list (Top 10 items). Table S2. Narcotic analgesics use list (Top 5 items). Table S3. Physical therapy use list (Top 10 items). (DOCX 20 kb) [file 13063_2018_3016_MOESM2_ESM.docx]

Additional file 1

The high rank lists of medication and physical therapy for non-specific neck pain, cervical disc herniation, and sprain and strain of neck were extracted from the 2014 National Patient Sample of the Korean Health Insurance Review & Assessment Service (HIRA-NPS)

Table S1. Non-narcotic analgesics use list (Top 10 items)

| Rank | Non-narcotic analgesics | Sprain/Strain of neck | Cervical disc herniation | Non-specific neck pain |
| --- | --- | --- | --- | --- |
|  |  | (%)^*^ | (%)^*^ | (%)^*^ |
| 1 | Aceclofenac | 25.36 | 30.06 | 25.49 |
| 2 | Diclofenac | 23.37 | 22.86 | 15.81 |
| 3 | Tramadol | 17.67 | 18.49 | 13.56 |
| 4 | Loxoprofen Sodium Hydrate | 17.63 | 15.89 | 13.65 |
| 5 | Talniflumate | 17.81 | 15.79 | 12.67 |
| 6 | Tramadol, combinations | 8.02 | 13.09 | 9.78 |
| 7 | Zaltoprofen | 4.35 | 4.38 | 3.87 |
| 8 | Chlorphenesin Carbamate | 3.95 | 2.91 | 3.15 |
| 9 | Meloxicam | 1.48 | 4.77 | 3.39 |
| 10 | Paracetamol | 2.79 | 1.87 | 1.98 |

^*^Percentage of patients using non-narcotic analgesics among total patients with indicated diseases.

Table S2. Narcotic analgesics use list (Top 5 items)

| Rank | Narcotic analgesics | Sprain/Strain of neck | Cervical disc herniation | Non-specific neck pain |
| --- | --- | --- | --- | --- |
|  |  | (%)^*^ | (%)^*^ | (%)^*^ |
| 1 | Pethidine | 0.16 | 1.03 | 0.35 |
| 2 | Codeine, combinations excl. psycholeptics | 0.14 | 0.56 | 0.35 |
| 3 | Remifentanil | - | 0.58 | 0.19 |
| 4 | Fentanyl | 0.01 | 0.38 | 0.14 |
| 5 | Oxycodone | - | 0.09 | 0.08 |

^*^Percentage of patients using narcotic analgesics among total patients with indicated diseases.

Table S3. Physical therapy use list (Top 10 items)

| Rank | Physical therapy | Sprain/  Strain of neck | Cervical disc herniation | Non-specific neck pain |
| --- | --- | --- | --- | --- |
|  |  | (%)^*^ | (%)^*^ | (%)^*^ |
| 1 | Superficial heat therapy | 71.79 | 75.12 | 65.17 |
| 2 | Deep heat therapy | 62.00 | 60.00 | 53.00 |
| 3 | Interferential current therapy | 36.07 | 37.45 | 32.05 |
| 4 | Transcutaneous electrical nerve stimulation | 31.16 | 32.30 | 28.20 |
| 5 | Intermittent traction therapy of the cervical spine | 5.12 | 46.23 | 17.60 |
| 6 | Laser therapy(Low power) | 6.64 | 7.10 | 7.47 |
| 7 | Simple therapeutic exercise | 2.44 | 4.04 | 3.44 |
| 8 | Myofascial trigger point injection therapy (≥ 2 points) | 0.64 | 1.45 | 1.38 |
| 9 | Paraffin bath | 0.71 | 1.64 | 1.03 |
| 10 | Myofascial trigger point injection therapy | 0.60 | 1.06 | 1.28 |

^*^Percentage of patients using physical therapy among total patients indicated diseases.
