# Supplementary material for: Comparative effectiveness and economic evaluation of Chuna manual therapy for chronic neck pain: protocol for a multicenter randomized controlled trial
Source: Trials. 2018 Nov 29;19:663. doi: 10.1186/s13063-018-3016-6 (PMC6267027; doi:10.1186/s13063-018-3016-6)
Supplement: Supplementary file 3 — Principal investigator (PI) and research physicians at main study site. (DOCX 20 kb) [file 13063_2018_3016_MOESM3_ESM.docx]

**Principal investigator (PI) and research physicians at main study site**

**Organization: Jaseng Medical Foundation**

**Contributions and** **role:** Principal investigators, original study design: In-Hyuk Ha, Joowon Kim, Min-young Kim, Hyun-Woo Cho, and Jae-Heung Cho

Organization of Steering committee and member appointment: In-Hyuk Ha, Yoon Jae Lee, Joowon Kim, Min-young Kim, Hyun-Woo Cho, and Jae-Heung Cho

Communication and exchange of opinion with PIs at each study site

Preparation of Institutional Review Board (IRB) documents and Case Report Form (CRF)

Trial management (random allocation, collection of adverse event data at each site, participant enrollment supervision, inspection and visits to study sites, budget allocation and management)

Data collection, quality control, monitoring, and analysis

**Steering committee (SC)**

**Organization and role:** All authors of this manuscript

**Contributions:** Protocol revision and decision on final protocol

Organization of Trial Management Committee and member selection

Designation of study sites for participant recruitment

Inspection of study progress, and decision on protocol revision, if needed

Decision regarding timing and method of study result publication

Determination of authorship in accordance with Authorship eligibility guidelines

**Trial Management Committee**

**Organization:** PIs and investigators at each clinical trial participant enrollment site

**Organization and role:** Submission of study protocol and obtaining IRB approval from pertaining IRB body at each study site

Clinical trial execution following protocol (e.g. participant recruitment, enrollment, data collection, CRF entry)

Collection and report of adverse events (AEs)
